# Achieving a hepatitis C cure: a qualitative exploration of the experiences and meanings of achieving a hepatitis C cure using the direct acting antivirals in Australia

**DOI:** 10.1186/s41124-018-0036-5

**Published:** 2018-08-04

**Authors:** Jacqueline A. Richmond, Jeanne Ellard, Jack Wallace, Rachel Thorpe, Peter Higgs, Margaret Hellard, Alexander Thompson

**Affiliations:** 1 Australian Research Centre in Sex, Health and Society, La Trobe University, Bundoora, Victoria Australia; 2 Disease Elimination, The Burnet Institute, Prahran, Victoria Australia; 3 Department of General Practice, The University of Melbourne, Parkville, Victoria Australia; 4 Kirby Institute, University of New South Wales, Kensington, New South Wales Australia; 5 Department of Public Health, La Trobe University, Bundoora, Victoria Australia; 6 Department of Epidemiology and Preventive Medicine, Monash University, Melbourne, Australia; 7 Department of Infectious Diseases, Alfred Health, Melbourne, Australia; 8 Department of Medicine, University of Melbourne, Melbourne, Australia; 9 Department of Gastroenterology, St Vincent’s Hospital, Melbourne, VIC Australia

**Keywords:** Hepatitis C, Cure, Qualitative, Lived experience

## Abstract

**Background:**

Universal access to the hepatitis C direct acting antiviral (DAAs) regimens presents a unique opportunity to eliminate hepatitis C in Australia. Large numbers of Australians have already been cured using these treatments, however, the numbers presenting for treatment have begun to plateau. This study explored how people experienced and understood being cured of hepatitis C, with the aim of informing interventions to increase uptake of DAA treatment among people with hepatitis C.

**Methods:**

This qualitative study used semi-structured interviews to explore the experiences of people with hepatitis C taking DAAs accessing both hospital and community clinics. Interviews were conducted 12 weeks after treatment completion. Participants were asked to reflect on their experience of living with hepatitis C, their reasons for seeking treatment, and their experience of, DAA treatments. Participants were also asked to reflect on the meaning of being cured, and how they shared this experience with their peers. Interviews were transcribed verbatim and key themes were identified using inductive thematic analysis.

**Results:**

Twenty participants were interviewed. While participants described a range of physical health benefits of achieving a hepatitis C cure it was an improved sense of psychological wellbeing that had the most significant impact on participants’ lives. The majority described their relief about no longer living with the burden of an uncertain future due to anxiety about developing liver disease or cancer, as well as fear of infecting others. Participants who had a past history of injecting drug use, described being cured as a way to break the connection with their past. Participants who were current injectors raised concerns about re-infection.

**Conclusion:**

Feeling “normal” and not infectious allows people to live with reduced psychological distress, in addition to the physical benefits of no longer being at risk of developing serious liver disease. Future engagement strategies targeting people who are not accessing hepatitis health care need to promote the lived experience of being cured and the substantial psychological, and physical health benefits, offered by achieving a cure. Interventions aimed at people who are currently injecting also need to highlight the availability of re-treatment in conjunction with primary prevention strategies.

## Background

The universal, government funded access to direct acting antiviral (DAA) treatments for hepatitis C in Australia presents a unique opportunity to eliminate hepatitis C. The Australian Government [1], in line with the World Health Organization (WHO) [2], has set the ambitious target of eliminating hepatitis C as a public health threat by 2030. During the first 15 months of access, an estimated 43,360 people initiated DAA treatment in Australia [3]. However, an estimated 200,000 Australians remain infected with hepatitis C and data suggests the number of people presenting for treatment in Australia plateaued in 2017 [3]. The decline in number of people accessing DAA treatment compared to 2016 reflects the clearing the of the so called “treatment warehouse” where patients were regularly engaging with clinical services while waiting for the DAAs, and highlights the importance of ongoing vigilance regarding testing and linkage to care. The reason for the plateau in number of people presenting for treatment is thought to be multifactorial: lack of knowledge about hepatitis C, [4, 5] the asymptomatic nature of the disease and the negative perceptions of previous interferon-based treatment mean that many people with hepatitis C do not prioritise the treatment [6]. Additionally, the social marginalisation related to current or past injecting drug use and the stigma and discrimination associated with injecting drug use, have been shown to act as significant barriers to accessing hepatitis C testing and treatment [7, 8].

Hepatitis C infection has been associated with multiple extrahepatic manifestations such as depression and diabetes [9], which can have a profound impact on quality of life [10, 11]. The clinical impact of achieving a sustained virological response (SVR) measured at 12 weeks after treatment completion, and now referred to as hepatitis C cure, [12] has been widely reported to reduce the risk of liver-related morbidity and mortality, [13, 14] and the risk of developing extrahepatic manifestations, [15–17] and is associated with improved quality of life [18]. From the patients’ perspective cure of hepatitis C is associated with improved energy levels, reduced uncertainty about the future, a more positive outlook on life, and improved personal relationships [19, 20].

While the public health approach to hepatitis C treatment focuses on population level elimination, [12] understanding the impact of cure for individuals and affected communities and communicating these experiences to people with hepatitis C and service providers is central in persuading others to access treatment services. This qualitative study explores experiences of being cured of hepatitis C; the findings contribute new knowledge to inform interventions to promote access to hepatitis C treatment.

## Methods

### Recruitment

Ethics approval for this study was granted by the St Vincent’s Hospital Melbourne, Low and Negligible Risk Human Research and Ethics Committee (LRR-209/15). Participants were recruited through one Melbourne metropolitan tertiary hospital-based liver clinic and three affiliated community-based clinics between October 2016 and April 2017. The three community-based clinics were located in inner and northern metropolitan Melbourne and staffed by medical specialists and nurses employed by the tertiary hospital to offer an outreach specialist service including access to hepatitis C testing, liver diseases assessment and DAA treatment. Nursing staff from each clinic invited consecutive patients who had achieved an SVR, 12 weeks after treatment completion to contact the researcher (JR), who provided a participant information statement and consent form. If the individual decided to proceed, written informed consent was obtained prior to the interview. Interviews were conducted by author JE and were conducted either face to face at the hospital liver clinic or on the phone. Interviews lasted between 20 and 45 min. Participants were offered a $AUD30 gift voucher after the interview.

### Data collection

Semi-structured interviews were conducted to explore the experiences of people who achieved an SVR after taking DAA treatment. Participants were asked to:

- Identify the barriers and enablers to treatment success from their perspective
- Explore the meaning of ‘cure’
- Describe the ways they shared knowledge and experiences of treatment within their peer networks
- Describe adherence strategies they used while taking the DAAs.

### Analysis

Interviews were transcribed verbatim and imported and coded in NVivo Pro, a qualitative data analysis computer software package. A thematic analysis was undertaken by authors JR and RT to identify text relating to the key themes associated with the research questions and informed the development of a coding framework. New themes identified after repeated close reading of the transcripts were added to the coding framework. Memos were used to record ideas about connections between the themes and to the overarching concept of cure.

## Results

### Participants

Twenty participants were interviewed during the study, with their characteristics presented in Table 1. Equal numbers of men (*n* = 10) and women (*n* = 10) were interviewed. Twelve participants attended the tertiary hospital liver clinic, with the remaining eight participants attending one of three community-based clinics. Participants reported being diagnosed with hepatitis C between two to twenty-seven years ago. Two participants reported being diagnosed with cirrhosis and a further two participants reported they had test results that indicated severe liver disease. Seven participants had completed interferon-based therapy prior to this course of the DAAs. For the remaining 13 participants, this course of the DAAs was their first hepatitis C treatment experience.

**Table 1:** Characteristics of participants interviewed for the study

Transcript	Gender	Year of diagnosis	Previous Interferon-based treatment	DAA Treatment location
1	Female	2005	No	Hospital
2	Female	2001	No	Community
3	Female	2007	No	Community
4	Male	2007	No	Community
5	Female	1997	No	Hospital
6	Male	2003	No	Community
7	Male	1997	Yes	Hospital
8	Male	2002	Yes	Hospital
9	Female	2015	No	Hospital
10	Male	2002	No	Hospital
11	Female	2010	Yes	Hospital
12	Male	1997	No	Hospital
13	Male	2013	No	Hospital
14	Female	2001	Yes	Community
15	Female	2005	No	Hospital
16	Male	2010	No	Community
17	Female	2000	Yes	Hospital
18	Female	1990	Yes	Hospital
19	Female	2012	No	Community
20	Male	1990	Yes	Community

### The meaning of achieving a ‘hepatitis C cure’

#### Psychological impact

Participants discussed their understanding of being cured in several ways. The most frequently identified outcome of achieving a ‘cure’ was being relieved of the psychological burden of living with hepatitis C, and this outcome was often prioritised over that of physical symptoms. Most participants reported experiencing few symptoms prior to commencing treatment, and as such, were often unaware of any physical burden of living with hepatitis C:

*It hasn’t changed my life that much because I never really had any symptoms… The biggest change is just…emotionally…you go from being someone with a chronic disease that has the potential to cut your life short to being free* (Participant 18, female).

Three participants were excited about not having to worry about their health and instead being able to plan for their future and old age, with a more positive outlook:

*It’s like a real weight has just been lifted from you. I probably believed that one day it would get me because you just think oh, liver cancer, it’s such an insidious nasty thing. If you get that, you’re buggered* (Participant 5, female).

#### Reduced uncertainty and fear

While some participants associated being cured with less uncertainty and fear about the future, others highlighted that being cured allowed them to clarify the physical effects of hepatitis C infection, which reduced their uncertainty related to their general health. Four participants noted that while they lived with hepatitis C, they were never sure whether to attribute illness or fatigue to their hepatitis C infection or another illness, or ageing. Since being cured, participants reported being more certain about the cause of other physical conditions or illnesses:

*It’s hard to tell what it’s actually doing to you and whether the fatigue or ill health is related to it because it was never very clear what* [hepatitis C] *did to you* (Participant 7, male).

Another participant described no longer worrying about whether symptoms were underlying signs of a more serious health condition:

*The fact that that’s not there anymore, like if I get sick or I’m ill means all I’ve got is that. I’ve just got the ‘flu ... It’s not that it’s my liver’s fricking packing up on me or something* (Participant 5, female).

#### “Feeling normal”

The experience of the cure also related to participants reporting they no longer felt infectious, particularly in the context of their family or people they lived with. This was often expressed in a marked shift in how they thought about themselves. For many the implied meaning of feeling “normal” was not being “dirty” or “infectious”:

*I feel normal again … I don’t feel different to everyone else (*Participant 6, male).

For some, no longer being infectious meant they could change their behaviour and personal interactions because they did not have to worry about transmission:

*At my house, with toothbrushes and razors and stuff, it’s been something that I’ve had to be really, really vigilant with, with children in the house and with my partner … it’s really nice to know that I don’t have that in me anymore, and there aren’t those risks there of passing it on to anyone else* (Participant 6, male).

However, one participant continued long term habits in avoiding physical contact to prevent possible transmission:

*I’ve got issues where I can’t kiss anyone or I won’t let anyone take a drag of my cigarette…I still can’t, because it’s been years like I’ve been doing that, so I still can’t stop doing that* (Participant 2, female).

One participant discussed the impact of cure on their interactions with health providers such as dentists and pathology collectors:

*I love not being infectious…That for me has probably been the biggest thing – not having to feel guilty every time I had a blood test done that I might infect somebody, going to the dentist is nowhere near as stressful* (Participant 17, female).

#### Breaking a connection to the past

Several participants who contracted hepatitis C through sharing injecting equipment, and who no longer injected, described a sense of guilt and shame about their past injecting; feelings which persisted years after they had stopped injecting. These participants explained that hepatitis C was an ongoing marker of their injecting history and that being cured broke that connection:

*It’s like the last mark … is now gone. All the rest is just memories that I don’t have to think about* (Participant 6, male).

Participants reflected on the ways being cured shifted their sense of self self-perception, and some felt that no longer having the disease would enable a break with the past:

*It does make me feel more normal in a community sense … that I don’t have appointments or doctors are going to find out or my daughter’s friends are going to find out. That’s very liberating* (Participant 2, female).

Others expected they would continue to experience guilt in relation to their past drug use:

*I still feel guilty about it…about having used drugs when I was younger, about having contracted the hepatitis C… It’s just a bit of a mental scar that I suppose I’m going to carry for a long time* (Participant 16, male).

#### Physical impact

While many participants had not felt they had physical symptoms before commencing treatment, some described experiencing improved physical health as a result of being cured. Three participants explicitly attributed their improved physical health to being cured of hepatitis C, while others were uncertain whether their perception that their health had improved was directly related to hepatitis C or a result of the psychological benefits that flowed from no longer living with the virus:

*I don’t know how much of that is psychological. I don’t think it’s all psychological. I’m sure there is a physical element … I have more energy, I am sleeping better, just a whole range of things* (Participant 16, male).

One participant with hepatitis C-related liver damage noted that even after being cured, she needed to undergo regular monitoring for liver disease. However, her concern about one day dying of liver disease was reduced:

*Because I am cirrhotic I still have to have regular FibroScans and liver function tests. In some ways those things haven’t changed. I think it is just more that my outlook has changed* (Participant 17, female).

For many participants the physical effects of living with hepatitis C only became apparent post cure. For those participants with significant liver damage, the ongoing need to monitor the liver for complications was a reminder of the physical damage inflicted by living with the virus for a long time.

### Adherence strategies

In addition to exploring the impact and meaning ascribed by participants to achieving a cure, the interviews explored the experiences of taking the DAAs. Overall, participants reported that adherence to the DAAs was easy and unproblematic. One reason cited for the ease of adherence was a strong desire to be hepatitis C free and a belief that inconsistent adherence to the treatment would affect their chance of being cured.

*I just made sure I did what the doctors recommended to beat this bloody thing* (Participant 3, female).

The most common adherence strategy described by participants was taking the tablet(s) at the same time every day, and either by establishing a new routine or fitting the DAAs into an established routine. Participants who were already taking daily medication commonly discussed incorporating the DAAs into this routine and taking the DAAs at the same time as the other medication, most often in the morning:

*In the morning with my contraceptive pill. As soon as I wake up … that’s usually what I do, the pill, brush my teeth, shower* (Participant 15, female).

Those who were not already taking medication discussed incorporating the DAAs into other daily routines:

*It was straightforward for me and I think that’s why it was good to do them at night. I had them by the bed and I’d have a drink of water and I’d plug my phone in … I built it into that kind of a routine* (Participant 5, female).

One participant, taking up to five DAA tablets a day, used a dosette box to support adherence while another used a calendar, and others mentioned checking each dose off on a chart that had come with the DAA packaging. Another who was not accustomed to taking daily medication recalled finding it difficult to remember to take the DAAs, and set a daily mobile phone alarm as a reminder.

Fourteen participants reported forgetting to take one or two doses, which in some cases made them feel extremely anxious as they were concerned it would influence the efficacy of the treatment. This episode motivated those participants to take the tablets as soon as they remembered:

*A couple of days I thought, you know, at lunchtime, oh, strewth, I haven’t taken my pills. (Laughs)… no, no, no, no. And I mean, it was so important. I mean, my future sort of rode on it* (Participant 18, female).

Other participants checked with their doctors regarding the recommended strategy for missed doses:

*I told the doctor and he said that’d be fine as long as I made sure that I kept everything, you know, spot on from then on, and I did* (Participant 6, male).

Overall, participants reported ease with adhering to the DAA regimen and developed individual strategies to support their daily administration.

### Barriers and enablers to taking DAA treatment

The challenges associated with using DAA regimens related to individual circumstances, such as pharmaceutical management of existing medical conditions and lack of awareness among health professionals of the DAAs. One participant discussed the need to cease taking existing medication to manage a mental health condition and this required closer monitoring while she was on the course of DAAs. Another participant replaced one medication with another as her liver enzymes had risen.

In terms of side effects, one participant reported experiencing high blood pressure from taking the DAAs, which caused side effects throughout the duration of the treatment:

*I still didn’t have an easy time because my specialist told me it would lower my blood pressure and it didn’t. It made my blood pressure go higher … it wasn’t fun for me at all* (Participant 14, female).

Inexperience and lack of confidence amongst health professionals regarding DAAs and potential drug interactions was highlighted by several participants as a potential barrier to access. One participant identified that their doctor was unwilling to prescribe the DAAs given their lack of knowledge:

*When I’d decided and rocked up to my appointment to ask her about it and she told me that she wasn’t prepared to prescribe because she hadn’t got her head around it, that was just a huge blow…if people aren’t even prepared to prescribe it, well, why are we doing this?* (Participant 19, female).

Another participant was disappointed about their doctor’s lack of understanding about potential drug interactions:

[A Nurse] *…told me about this website from … a hospital that listed all the interactions of the hepatitis C drugs with existing drugs. I’d gone right through that and checked what I was taking against all that and that was how I realised that I shouldn’t actually be taking* [the medication]*. So I spoke to my GP about it and we sorted that out … It was really only the fact that* [the nurse] *was so on the ball that we ended up getting it sorted out* (Participant 16, male).

However, most participants reported being supported by health professionals during treatment. One participant, who was currently injecting drugs discussed difficulties in treatment adherence as their life was “a bit chaotic”, and described how the health professionals in a community-based service supported adherence.

Another participant discussed that the anticipation of side effects was a barrier to their commencing treatment; the fear of potential side effects was worse than the experience.

*I approached it with a degree of trepidation but as it turned out I just simply got stuck into it and had very minimal side-effects* (Participant 5, female).

Most participants were treated through specialist services either in a tertiary hospital or in an outreach clinic in the community; only three participants were prescribed their DAA course by a General Practitioner (GP). As interviews were conducted at the beginning of the implementation phase of the DAAs in Australia when few GPs were involved in prescribing DAAs and awareness raising activities had not officially commenced, the experiences of participants with GPs may have been be less than ideal.

### Sharing knowledge with peers

All participants reflected on their positive experiences of the treatment and being cured by recommending that people with hepatitis C seek advice from their doctor about taking DAAs:

*I have told a few people and other people have asked me … I recommend it* (Participant 1, female).

*I’m reasonably medically aware I suppose and cautious, but with that background I would urge anybody with hep C to very much be confident about getting onto this drug to cure themselves* (Participant 12, male).

While all participants emphasised the positive aspects of being cured, some also thought that it was important to inform prospective patients of the potential side effects:

*The only thing was that I really would be reluctant to tell people that these medications are side-effect free … there’s a 2% chance that anyone would have the reaction I did* (Participant 17, female).

One participant said that although they would like to share their experiences of being cured with peers and the broader community, they remained cautious about telling people about their experience of living with hepatitis C given the stigma associated with contracting the virus through injecting drugs:

*One part of me wants to sort of yell it at the top of my voice and say, look, this is an amazing, miraculous cure out there. Please, anyone that’s got hep C, go and get involved with it. But then the other part of me, sort of I suppose the conservative – but … I still feel guilty about it* (Participant 16, male).

There was a strong sense that the DAAs needed to be distinguished from interferon-based treatments with participants emphasising the need to advertise that DAAs were more effective and had fewer side effects than interferon-based treatment, and were simple to take:

*I’d tell them, no matter what your experience with past drugs … this is totally different* (Participant 18, female).

The two participants who identified as current injecting drug users were positive about their treatment experience and said they would encourage peers to seek treatment. One participant shared her experiences formally as a peer educator:

*I’d already started working as a peer educator, so part of that was an obligation to other users and needing to know what I was trying to tell people and encourage people to do... if I’m going to tell another user to do something I want to at least have experienced it, so I know what the hell I’m telling them* (Participant 19, female).

While the current injectors were positive about taking the DAAs, they identified potential barriers facing other people who inject, including the potential for re-infection. One participant was concerned that re-infection would become a new source of stigma:

*I think that there’s also something to be said about the stigma and the guilt that goes along with re-infection. That’s a whole new monster that we’re going to have to deal with soon* (Participant 19, female).

This same participant also proposed that some current injectors would continue to hold off on treatment because they conceptualised treatment as something to embark upon after they finished injecting drugs:

*I am finding that just even talking to the community on the street, it’s just like - yeah, big deal, there’s a treatment, but I’m not done with the life at the moment here, you know. And they do see it as something to happen when they’ve done with their using life* (Participant 19, female).

## Discussion

There is no disputing the revolutionary impact the DAAs have had on the clinical management of hepatitis C. While most participants involved in the current study had not experienced symptoms related to hepatitis C, they described feeling lucky to have escaped the infection relatively unscathed. Most significant was the psychological impact of being cured, highlighting the serious impact of hepatitis C and drug use related-stigma on the lived experience of people with hepatitis C and the uncertainty related to the physical impact of living with the infection.

Participants no longer felt infectious or a threat to their families and friends in terms of onward transmission, and for several participants, the benefit of breaking a link to previous drug use was transformative. This experience is echoed by the findings of Harris (2017) who describes hepatitis C treatment as promising social reconnection, social redemption from the drug using past and a return to ‘normality’ [21]. While most participants were not currently injecting drugs, the label of “drug user” inferred by a diagnosis of hepatitis C was an important motivator to access treatment. Also, the relief of no longer needing to worry about the risk of illness or death because of an infection contracted during their youth was immensely important.

Participants were passionate advocates for sharing the benefits of being cured with people in their network and complete strangers. The unexpected result of feeling “normal” because they were no longer infectious was highlighted as an important message to communicate to people not currently engaged in hepatitis C health care. The psychological relief, in addition to the physical benefits of no longer being at risk of developing serious liver disease and cancer were perceived as positive outcomes of being cured. However, while participants were keen to share their cure stories with peers and the communities, they also articulated a continuing fear of “outing” themselves as someone who contracted a disease most prevalent among people who inject drugs. As discussed, this label can have severe social ramifications such as alienation and discrimination that result in poor access to health services, [22, 23] and the ongoing fear of stigma created a barrier to sharing the good news.

In Australia, the number of people initiating DAAs has plateaued from extremely high volumes in 2016 to a lower but constant number during 2017. Although evidence suggests that Australia has a high diagnosis rate of approximately 80%, [24] the quality and understanding of the meaning of the diagnosis is unknown. Increasing testing rates and subsequent engagement in treatment is essential if hepatitis C elimination is to be achieved. Hence it is vital to engage and re-engage people with, or at risk of hepatitis C, many who were given outdated information decades ago or were frightened by the side effects associated with interferon-based therapy. Re-engaging people with or at risk of hepatitis C requires a committed health system response and knowledgeable and engaged health professionals, as well as directly promoting the experience of people with recent treatment experience. A first step could be a systematic testing campaign where the benefits of cure negate the potential fear of stigma and discrimination associated with hepatitis C. Integrating and normalising hepatitis C treatment and care in the primary care setting with systematic programs that offer testing to all patients attending the practice regardless of risk factors is one possible strategy [25].

In the current study, 13 participants were naïve to previous hepatitis C treatment, including interferon-based regimens. These participants were among the first group treated in Australia and sought DAA treatment quickly; two participants reported being forced to commence the DAAs early due to the imminent risk of illness resulting from severe liver disease. The desire to physically remove the virus and reduce their risk of morbidity and mortality was the predominant motivator to accessing the DAAs, however, the psychological impact of being cured was significant.

One barrier to accessing DAAs noted by several participants was the lack of knowledge and awareness among health professionals about hepatitis C and the DAAs. Twelve participants accessed DAAs through a tertiary hospital clinic because they had all been attending the clinic for several years for ongoing surveillance and monitoring of their liver health. Whereas the eight participants recruited from a community-based clinic had sought out the clinics for the purpose of accessing DAAs, although technically they should have been able to access them through any GP. It is worth noting that this study was conducted during the early DAA implementation phase in Australia; while the initial proportion of DAA prescriptions initiated by primary care practitioners was low (8%), it steadily increased over time to 39% in June 2017 [3]. Therefore, while participants were all able to find health services or practitioners that could facilitate access to the DAAs, this was not a universally straightforward at the time the interviews were conducted. Relying on individuals’ personal motivation to access DAAs is not a sustainable response. There are many workforce development projects underway in Australia, that aim to build the capacity of primary care practitioners and ensure that people with hepatitis C have multiple access points to DAA treatment; these programs need to be sustained overtime.

Future promotion of the next wave of DAA candidates in Australia should draw on the firsthand experiences of people who have been through the DAA treatment and have achieved cure, including people who currently inject drugs. Roger’s ‘Diffusion of Innovations’ theory seeks to explain how, why, and at what rate new innovations (ideas, technology and/or products) gain momentum and diffuse or spread through a specific population. This theory suggests that when an innovation, in this case, DAAs, are promoted by respected individuals within a social network, it creates a desire to adopt the innovation [26]. This perspective is supported by research that shows that people with hepatitis C have played an important role in engaging people who inject drugs with hepatitis C in treatment services [27–30].

The limitations of this study should be acknowledged when interpreting the data; the small sample size and inherent limitations regarding the generalisability of qualitative data at a population level and the timing of recruitment during the early stages of the DAA implementation in Australia could all affect the relevance of this data to the current cohort of people seeking DAA treatment. Study participants most likely represent early adopters of the DAAs, who sought out the treatment because they were well informed and motivated, compared to people currently accessing treatment who have preferred to wait and gather evidence of the DAA’s success. The environment, in terms of the public’s and health workforce’s awareness has most likely improved over time, making access to treatment less reliant on highly motivated individuals. The focus of this study was on participants’ experiences of being cured, therefore, demographic information about participants’ age, mode of infection and liver disease staging were not systematically collected. The authors acknowledge that these factors may influence people’s experience of being cured of hepatitis C.

Most participants in this study found DAAs easy to take with generally mild and manageable side-effects. Nonetheless they indicated it was important to acknowledge the potential for DAA side effects, as this would enable people to plan and establish contingency plans for the treatment course and support adherence. Overall, adherence to the DAAs was not an issue of concern, in this study, participants described how they identified their own memory hooks and existing routines to support the daily administration of the DAAs [31]. However, these participants were particularly motivated to achieve a cure. In the future, more planning and support may be needed to support adherence for people who are more sceptical about the DAAs and of their own need to achieve a cure.

The perspectives of participants who currently inject drugs illustrates that the availability and effectiveness of DAAs are not the only factors that influence their decision to start treatment, which is supported by Goutzamanis and colleagues (2016) [32]. Health promotion messages need to shift the perceptions of current injectors around treatment and re-treatment to ensure they understand that DAAs are universally available and people who currently inject are a priority population for treatment access. In providing hepatitis C treatment and care, clinicians, pharmacists and other service providers should highlight the individual health benefits of re-treatment and the population level benefits of stopping onward transmission. The issue of re-infection also demonstrates the importance of promoting ongoing hepatitis C testing for current injectors and for continuing investment in harm reduction programs including needle and syringe programs.

## Conclusion

Future engagement strategies targeting people with hepatitis C who are not currently accessing DAA treatment need to use the lived experience of being cured, particularly the significant psychological impact such as relief about no longer being a transmission risk for family members. Promoting this alongside the health benefits offered by the hepatitis C cure, and having a reduced risk of developing liver disease and liver cancer are crucial in supporting elimination efforts.
